# Peripheral Arterial Disease: A Narrative Review

**DOI:** 10.7759/cureus.40267

**Published:** 2023-06-11

**Authors:** Divya Parwani, Mohamed A Ahmed, Anmol Mahawar, Vasavi Rakesh Gorantla

**Affiliations:** 1 Anatomical Sciences, St. George's University School of Medicine, St.George's, GRD; 2 Anatomical Sciences, St. George's University School of Medicine, St. George's, GRD; 3 Anatomical Sciences, St. George's University of Medicine, St. George's, GRD

**Keywords:** pad management, endovascular advancements, duplex ultrasound, atherosclerosis, peripheral arterial disease

## Abstract

Peripheral arterial disease (PAD) describes the partial or complete occlusion of blood flow in the distal arteries of the body. A decreased arterial patency may occur due to a reduction in the elasticity or diameter of the vessel. The goal of interventions is to decrease incidence and reduce complications by identifying and minimizing the primary causes. This paper discusses PAD affecting the aortoiliac, common femoral, and femoropopliteal arteries. In a significant portion of the population, PAD may lack usual symptoms such as limb pain, claudication, and diminished pulses. Imaging techniques become crucial to ensuring timely diagnosis, monitoring treatment effectiveness, and preventing recurrence. Duplex ultrasound (DUS) is a cheap and non-invasive preliminary technique to detect atherosclerotic plaques and grade arterial stenosis. Magnetic resonance angiography (MRA) provides the added advantage of minimizing artifacts. Digital subtraction angiography (DSA) remains the gold standard for grading the degree of stenosis but is only employed second-line to DUS or MRA due to the high dose of nephrotoxic contrast. Computed tomography angiography (CTA) is able to overcome the anatomical limitations of DUS and MRA and proves to be a suitable alternative to DSA in patients with renal disease. Preventative measures involve monitoring blood pressure, cholesterol levels, and tobacco usage. First-line treatment options include endovascular procedures as well as surgical interventions in cases of significant arterial involvement. Endovascular treatments involve the use of balloon angioplasty, drug-coated balloons, and drug-coated stents, to name a few, that serve as minimally invasive techniques to manage PAD. Surgical procedures, although more complex, are considered gold-standard treatment options for long and intricate lesions. Endovascular methods are generally preferred over surgical options as the complication risk is severely reduced and the rates of reintervention are comparable to surgical options.

## Introduction and background

Peripheral arterial disease (PAD) results from the isolated and/or combined loss of arterial elasticity, pathological platelet aggregation, and increased incidence of microvascular complications, as seen in cases of hypertension, hyperglycemia, hyperlipidemia, hypercholesterolemia, and smoking [1]. Uncontrolled, long-standing hypertension lowers the vasodilatory effects of endothelial cells and activates the renin-angiotensin-aldosterone system (RAAS), which leads to hyaline atherosclerosis and loss of arterial elasticity [2]. Type II DM has been linked to PAD through WBC-induced endothelial dysfunction [3]. Smoking causes decreased vasodilation and inappropriate platelet aggregation, leading to atherosclerosis and thrombotic events [4]. Long-term complications of PAD include ischemia and present as absent pulses, cyanosis, nonhealing ulcers, and intermittent claudication [5]. It may progress to critical limb ischemia (CLI), sepsis, amputation, and death [6].

The objectives of medical intervention are to promote preventative measures in the population, detect disease in its early stages, manage symptoms appropriately, and prevent recurrence and mortality. Artery disease in the aortoiliac, common and external iliac, femoral, popliteal, anterior and posterior tibial, and dorsal pedal arteries is covered. Common arterial mapping techniques such as duplex ultrasound (DUS), magnetic resonance angiography (MRA), digital subtraction angiography (DSA), and computed tomography angiography (CTA) are analyzed for their effectiveness in visualizing different arteries. Non-invasive methods such as smoking cessation, antithrombotic therapy, and statin therapy have been researched for the management of the patient. Endovascular and surgical interventions have also been reviewed. After analyzing various pieces of literature, this article aims to elaborate on the pathophysiology of PAD as well as early detection and appropriate management of the disease.

## Review

Pathophysiology

Hypertension is one of the leading causes of atherosclerotic plaque formation [1]. While hypertension can be classified into two broad types, its association with endothelial cells is thought to remain the same. The interaction of high blood pressure with endothelial cells is considered a two-way procedure. It is observed that endothelial dysfunction decreases nitric oxide synthesis, leading to decreased vasodilation and ultimately hypertension [2]. Another blood pressure regulator, such as RAAS, plays a major role in maintaining blood pressure. While the objective of RAAS is to optimize blood pressure levels, the system can be maladaptive in nature, contributing to arterial stiffness due to endothelial dysfunction [3].

Effects on microcirculation due to hyperlipidemia, diabetes mellitus, and hypertension

Apart from arteries and veins, the remaining vasculature lacks the three layers of smooth muscle: tunica intima, tunica media, and tunica adventitia. Capillaries contain pericytes, which line the endothelial cells and form an interrupted wall, allowing for gas exchange and nutrients. Pericytes secrete the growth factor angiopoietin-1, which binds to the Tie-2 receptor found on endothelial cells [2]. This interaction allows for the release of anti-apoptotic factors such as BAD and procaspase-9 as well as survival factors such as Survivin [2]. Factors such as reactive oxygen species (ROS), hypoxia, and neoplasia negatively impact microcirculation. The interplay of cholesterol, diabetes mellitus, and high blood pressure with pericytes has been discussed separately. Hypercholesterolemia causes the downregulation of pericytes due to decreased expression of N-cadherins as well as a reduction in NO synthase, an important regulator needed for the recruitment of pericytes [2]. Downregulation of growth factors such as vascular endothelial growth factor (VEGF) and upregulation of leukocytes due to ROS are also seen in hyperlipidemia [7]. Chronic hyperglycemia is also known to affect pericyte loss, leading to endothelial cell damage resulting in hypoxia and ultimately neovascularization. This in turn leads to diabetic retinopathy (DR), one of the first complications of type 2 diabetes mellitus (T2DM) [8]. As opposed to hyperlipidemia and T2DM, arterial hypertension results in the proliferation of pericytes. This is due to the overexpression of growth factors such as fibroblast growth factor-2 and interleukin-6 [9]. Despite an overproliferation of pericytes, the end result is capillary dysfunction. Microvascular complications further complicate the patient profile for PAD.

Smoking

In addition to coronary artery diseases and myocardial infarction, cigarette smoking has shown a high correlation with arterial atherosclerosis. In their study, Walsh et al. [10] found that heavy smokers and former heavy smokers have a significantly increased risk of developing superficial femoral artery atherosclerosis. In addition, passive smoking increases the risk of coronary artery diseases; smoking exerts its effect through cyclical short- and long-term mechanisms. The short-term mechanism involves the increased aggregation of platelets, contributing to the inflammatory response that leads to atherosclerotic plaque formation [4]. Prior to vascular structural changes, inflammatory cellular modifications precede atherosclerotic formation, which includes declining contraction of the vessels [11]. Smoking products such as benzo(a)pyrene injure the endothelial cells, exposing subendothelial factors and promoting atherosclerotic plaque formation [4].

Diagnostic methods

PAD is a severe disease that has lifelong implications for patients, which makes imaging modalities extremely important in catching the disease early, providing appropriate treatment, and preventing recurrence. Imaging techniques such as DUS, MRA, DSA, and CTA are excellent and comprehensive tools in the diagnosis of arterial diseases. Symptomatically, PAD presents as exertional leg pain, a diagnosis of claudication, absent distal pulses, dependent rubor, and a nonhealing ulcer [5], which may progress to regressed wound healing, progressing infection, sepsis, amputation, and death [6]. The importance of imaging is highlighted in a study where DUS results varied from symptoms like pulsation (femoral artery [25%] and popliteal artery [34%], respectively) [6]. On imaging, the arterial disease presents with a lipid-rich necrotic core (LRNC), intraplaque hemorrhage (IPH), calcification [12], intermittent claudication (43.5% [13] to 65.4% [14]), chronic limb-threatening ischemia (CLTI) (56.4%), rest pain (26.5%), foot ulcers, or ischemic gangrene. A greater than normal (1.0-1.4) ankle-brachial index (ABI) is a strong indicator of loss of elasticity, as seen in calcification and stenosis [15]. The proximal superficial femoral artery is the most common (41.1%) site of femoral artery stenosis [14], with the common femoral artery (CFA) being the second most common. The most common site of compression of the popliteal artery is at the head of the gastrocnemius muscle [16]. The anterior tibial artery (ATA) is most commonly damaged/occluded in the distal ⅔, leading to dorsal pedal artery ischemia and redistribution of blood through the posterior tibial artery (PTA) anastomosis [17].

DUS is a superior vascular mapping tool because it is cost-effective and eliminates complications associated with iodine contrast and ionizing radiation, especially in patients with renal disease [18]. DUS is a valuable tool to detect lipid-rich plaques [12], luminal stenosis, and arterial wall thickness. It can also be used to diagnose focal intra-stent stenosis [19]. DUS characterizes hemodynamics with the help of the parameter: peak systolic velocity (PSV) [20], which is used to grade stenosis, where a PSV ratio >2.4 [5] is an indicator of severe stenosis.

In their article, Baril et al. suggested a PSV of ≥275 cm/s and a velocity ratio, Vr, ≥3.50 to be highly suggestive and predictive of ≥80% stenosis [21]. Employing DUS to follow up after endovascular repair yields no significant difference against CTA (R=0.97, P<0.001) [22]. In their study, d'Audiffret et al. compared the accuracy of DUS and CT in detecting endoleaks and assessing abdominal aneurysm diameters. The study resulted in a correlation of 0.89 (P < 0.001) between DUS and CT. In comparison to CT, DUS had low accuracy reports, with cases ranging from 5.6 mm smaller to 11.6 mm bigger than the reported diameter on CT [23]. Moreover, DUS underestimates the degree of stenosis compared to CTA (degree of concordance has been reported in more studies [5,24] between DUS and CTA: common iliac: 0.8 (0.71-0.88), right external iliac: 0.84 (0.76-0.92), left external iliac: 0.71 (0.61-0.81)) or MRA (degree of concordance between DUS and MRA: 86%). DUS provides a moderate to strong correlation with MRI [12]. In aortoiliac segments, DUS demonstrated 90% sensitivity, 85.0% specificity, 89.6% positive predictive value (PPV), 85.4% negative predictive value (NPV), and 88% accuracy [25]. To map the femoral arteries, the patient is positioned supine, and the procedure is repeated with the hip laterally rotated [20]. In native arteries, DUS had a PSV = 180 cm/s, 80% specificity, and 66% sensitivity for >50% stenosis [20]. There is a strong degree of concordance between DUS and CTA in the right femoral (0.82 [0.69-0.95]) and left femoral (0.88 [0.76-1]) arteries [24]. In the femoropopliteal segments, DUS provided 94.8% sensitivity, 80.2% specificity, 94.1% PPV, 89.0% NPV, and 90.1% accuracy [25]. The best hemodynamic parameter to assess femoropopliteal stenosis is proximal PSV (>50%: proximal PSV=2.6, >70%: proximal PSV=3.3, >80%: proximal PSV=3.9) [26]. To map popliteal arteries, the patient is positioned supine, and the studies are repeated in a prone position with the knee flexed at 15 degrees [27]. The insolation angle between the flow of blood and the DUS beam was kept at >60 degrees [20]. Dynamic maneuvers may lead to flow changes and false positives in the case of the popliteal artery. PSV >200 denotes significant stenosis [27]. The limitations of using DUS are obesity, abdominal bloating (inadequate bowel preparation), calcification of the common iliac in aortoiliac segments [28], and IPH due to the vulnerability of atherosclerotic plaque in all arteries [12].

MRA is an excellent technique to visualize and grade arterial stenosis. However, its use is limited due to contraindications with intravascular stents [16,29], implanted cardiovascular devices, metallic foreign bodies, pacemakers, cochlear implants, metallic vascular clips, and metallic dental retainers. The contrast medium and ionizing radiation used in MRA cause renal disease. Using IV iron or ferumoxytol as a contrast medium can cause mild adverse effects in patients with kidney failure. MRA proved to be better for mapping calcified arteries by minimizing artifacts compared to CTA [30]. This diagnostic advantage is especially helpful in diabetic patients with chronic limb ischemia, where MRA was at par with CTA [31]. Cine-fast interrupted steady-state in combination with arterial spin labeling (cFASL) MRA assesses blood flow velocity by measuring the displacement of a radiofrequency labeled bolus of arterial spins, yielding high-resolution flow patterns similar to DSA. A comparison of two MRA techniques, quiescent interval single-shot MRA (QISS-MRA) [32] and contrast-enhanced MRA (CE-MRA), showed that QISS-MRA was useful to visualize ulcers, infections, and runoff vessels in end-stage PAD in diabetics, but a longer duration presented more artifacts. QISS-MRA is safer in patients with kidney disease and in patients with contraindications to gadolinium and iodinated contrast. MRA demonstrated 87.5-100% sensitivity and 76.5-98.5% specificity in native arteries with >50% stenosis [33]. DSA validated MRA results in femoropopliteal segments (>95% correlation), but overestimated the degree of stenosis [34]. In Buerger’s disease, MRA and DUS were found to be more accurate diagnostic modalities than CTA. MRA provided 40% longitudinal coverage with white matter lesions (WMLs) visualization (severe → >3 (31.3%, P = 0.003)) [12]. MRA was not found to be useful in visualizing the anatomy of the popliteal fossa due to the gastrocnemius muscle being sensitive to motion artifacts caused by a longer duration of the procedure [35].

DSA is the gold standard for visualizing arterial disease over CTA and MRA [17]. DSA requires a long fluoroscopy time (734.4 s) and higher doses of contrast volume (112.9 ml) and radiation dose (21.84 Gy/cm2) [13]. Two techniques of DSA, proximal radial (PR-DSA) and distal radial (DR-DSA), were compared [14]. PR-DSA requires dual access and has a high incidence of minor vascular complications; however, PR-DSA has low bleeding complications, a lower cost, and easy cannulation for follow-up management procedures for visualizing the patency of the arterial lumen. DR-DSA provides a less invasive, superficial subcutaneous tissue approach that may result in pseudoaneurysms and hematomas. The DR-DSA catheter is smaller and angled but longer than the PR-DSA. Another technique, spin DSA, is extremely useful in patients with severe angulation or severe gantry angles [36]. The limitation associated with DSA is its invasiveness involving ionizing radiation, and it is often used as a second line to inconclusive MRA or DUS [16]. In some cases, inadequate visualization of the arterial lumen may lead to underestimation of stenosis and may lead to undertreatment and/or recurrence of vascular diseases [37].

CTA is another excellent technique to provide high-resolution tissue contrast imaging in vascular diseases. CTA employs iodinated contrast like DSA but proves to be less invasive and has a lower risk of renal disease from contrast and radiation [38]. It can still be used in patients with renal disease if the benefits outweigh the risks [39]. In aortoiliac segments, CTA is a reliable, less invasive, yet equally effective alternative to DSA [40]. In arteries with calcification, CTA is less advantageous to MRA in being able to provide clear mapping with no artifacts [30]. In the popliteal artery, CTA is able to overcome the anatomical limitation of the popliteal fossa (gastrocnemius muscle), as seen in DUS and MRA [27]. In the tibial artery, CTA shows high-resolution three-dimensional anatomy but falls short in visualizing hemodynamic parameters [17]. Therefore, DSA remains the gold standard for diagnosing arterial disease in lower limb arteries.

Management

With an increase in the aging of the population as well as a surge in risk factors such as tobacco smoking, hypertension, and diabetes mellitus, there is a continual rise in the manifestation of PAD in the population. Smoking cessation, supervised exercise therapy, lipid-lowering agents, and antihypertensives are shown to have beneficial effects in delaying disease progression and providing symptomatic relief [41].

In our research, tobacco usage was found to be a significant risk factor for the development of PAD. There is innumerable evidence to suggest a worsening of PAD due to tobacco smoking. Patients with symptoms of PAD should be asked about their smoking habits, and interventions must be adopted to make improvements in their lifestyles. Smoking cessation methods must be incorporated. Frequent questioning of the patients' smoking habits at every medical encounter as well as counseling about effective alternative methods such as nicotine replacement therapy (NRT), varenicline, and bupropion should be included [9,42,43].

NRT such as lozenges, patches, and gums are effective in reducing withdrawal symptoms as they replace tobacco with other sources of nicotine and are effective in curbing withdrawal symptoms and cravings until the patient is able to culminate their smoking habits. Varenicline, a partial acetylcholine nicotinic agonist, works by increasing dopamine levels, thereby reducing cravings and withdrawal symptoms. Bupropion is thought to be beneficial in the management of PAD. It works by preventing the reuptake of norepinephrine and dopamine, providing stimulatory effects to the brain [9,42,43].

Supervised exercise therapy is an essential, non-invasive method for managing the symptoms of PAD. Walking is shown to increase nitrites and, as a result, improve perfusion. McDermott summarized in their findings that people with PAD who underwent supervised treadmill exercise indicated an improvement of around 180 m more than people with no exercise. Home-based exercise was also effective in improving the disease [44].

Antithrombotic therapy is beneficial for patients with atherosclerotic disease. Alonso-Coello et al. found that single antiplatelet therapy should be recommended for the prevention of cardiovascular events in patients with symptomatic and asymptomatic PAD [45]. The Society for Vascular Surgery also noted similar findings, emphasizing the need to manage atherosclerotic cardiovascular outcomes [46]. However, in a study evaluated by Eikelboom et al., 27,395 participants with stable atherosclerotic cardiovascular disease were subjected to a double-blind trial where subjects were randomly assigned to take aspirin (100 mg once daily) along with rivaroxaban (2.5 mg twice daily), rivaroxaban twice daily (5 mg), or aspirin (100 mg once daily). It was concluded that the group taking aspirin (100 mg once daily) plus rivaroxaban (2.5 mg twice daily) had better health outcomes compared to monotherapy using aspirin (379 patients [4.1%] vs. 496 patients [5.4%]; hazard ratio, 0.76; 95% confidence interval [CI], 0.66 to 0.86; P<0.001; z = −4.126). The dual therapy, however, has a higher risk of bleeding compared to aspirin alone [47].

All patients with atherosclerotic cardiovascular disease should be on statin therapy, regardless of their baseline LDL range. In their study, O'Donnell et al. found that patients who underwent statin therapy according to the American Heart Association guidelines of 2013 exhibited fewer major adverse limb events (MALE) (HR, 0.71; 95% CI, 0.51-0.97; P < 0.05), lower rates of revascularization, and lower mortality rates (HR, 0.73; 95% CI, 0.60-0.99; P < 0.05) [48]. Furthermore, Bonaca et al. found in their study that the PCSK9 inhibitor evolocumab was effective in reducing LDL cholesterol and preventing MALE in patients with PAD [49].

Hypertension is one of the major causes of atherosclerosis; however, the relationship between a reduction in blood pressure and the manifestation of PAD has yet to be explored [50]. That being said, the aim should always be to optimize blood pressure levels to reduce morbidity and mortality [51]. There is insufficient research on the interplay of glucose with the manifestation of PAD; however, patients with T2DM should aim to have an HbA1C level <7% [52].

Invasive methods

Endovascular Revascularization

Table 1 summarizes the advantages of endovascular methods.

**Table 1:** The advancements of endovascular methods.

	Author	Country	Design and study population	Findings	Conclusion
1.	Eleissawy et al. [53]	Egypt and Belgium	Prospective cohort study (n=53)	14.84% (n = 53) who underwent endovascular treatment showed 92% limb salvage compared to 88% in the surgical group (P = 0.564). The endovascular group showed a lower length of stay compared to the surgical group. (6.24 ± 0.37 vs. 1.84 ± 0.19 days, respectively, P = 0.001).	The endovascular group showed lower length of hospital stay compared to the surgical group, thereby resulting in lesser complications. Limb salvage rates are higher in the endovascular group. Overall, endovascular interventions have comparable patency rates and lesser long term complications.
2.	Nordanstig et al. [54]	Sweden	Prospective randomized control study (n=158)	50% patients (n = 158) were assigned to invasive treatment strategies (INV). There was a notable difference in the intermittent claudication distance (ICD) when INV was compared to non-INV management (INV = +124m vs Non INV= +50M p = 0.003). Overall vascular quality of life (VascuQoL) also suggested results favoring INV methods (P ≤ 0.001).	Overall improvements were observed in patients who underwent INV when compared to non-invasive methods. INV strategies provided better long term outcomes and patients with ICD showed guaranteed improvements with revascularization.
3.	Caradu et al. [55]	UK	Retrospective non-randomized trial (n=255)	48% (n = 255) patients underwent endovascular treatment using 2D fusion imaging. The injected contrast medium was significantly lower compared to the control group (34.7 ± 13.8 mL vs 51.3 ± 26.7 mL; P < 0.001) and dose-area product (8.9 ± 9.9 Gy/cm^2^ vs 13.5 ± 14.0 Gy/cm^2^; P = 0.003).	With advancements in endovascular interventions, an overall improvement in the quality of life can be expected in the patient. The use of 2D fusion imaging has caused >30% reduction in the radiation dose and contrast dye needed, which denotes the significance of overall evolution of endovascular methods.
4.	Fakhry et al. [56]	Netherlands	Randomized clinical trial (n=212)	106 patients (n = 212) who underwent combination therapy (ER + SET) exhibited better outcomes than patients only on SET (from 117 m to 1237 m for an improvement of 1120 m vs. from 135 m to 847 m for improvement of 712 m, respectively) (mean difference, 408 m; 99% CI, 195-622 m) as well as combination therapy showed better VascuQoL outcomes compared to SET (1.34 [99% CI, 1.04-1.64] in the combination therapy group vs 0.73 [99% CI, 0.43-1.03])	Patients with ICD show comprehensive QoL improvements when SET is combined with ER. An integrated approach in the management of symptoms has proven to show better outcomes.
5	Totić et al. [57]	Bosnia and Herzegovina	Prospective study (n=80)	Patients with CLI and TASC II C and D lesions were followed for a period of 28 months. Overall survival rates in patients who sought surgical and endovascular treatment did not show significant differences (100% vs. 97.5%; p=0.317), respectively.	Endovascular treatments are preferred over surgical interventions as they are less invasive
6	Pacha et al. [58]	USA	NIS database (n=394,504)	56.1% patients (n = 394,504) patients underwent ER and showed lower in-hospital mortality rates compared to the surgical group (3.0% vs. 5.8%, adjusted OR: 0.61 [95% CI: 0.58 to 0.63]) as well as lesser leg amputations compared to the surgical group (3.3% vs 4.1%, adjusted OR: 0.77 [95% CI: 0.74 to 0.79]).	Octogenarian patients showed better cardiovascular and limb outcomes when treated with ER compared to surgical treatments.
7	van den Hondel et al. [59]	Netherlands	Systematic review	A total of 13 articles were reviewed and it was deduced that endoprosthesis had similar outcomes compared to surgical bypass (31% vs. 55%, P = 0.048, respectively) as well as fewer complications.	With new technological advancements in endovascular procedures, overall outcomes of patients are comparable with surgical procedures, with fewer obstacles.
8	Cannavale et al. [60]	UK and China	Retrospective study (n=32)	32 vessels were treated, of which 21 lesions 65.6% totally occluded. Recanalization with guidewire was successful in 71% (21 lesions) and usage of catheter alone yielded 29% (9 lesions) successful findings.	Demographic observations show that patients with complete total occlusions (CTO) of vessels usually are elderly and have comorbidities such as diabetes and hypertension, making surgical options unsafe. Endovascular procedures such as cross-catheters help in treating CTOs without significant problems.
9	Schillinger et al. [61]	Austria	Randomized controlled trial (n=104)	51 out of 104 patients underwent primary stent therapy and saw the rate of restenosis at 6 months was 24%. Additionally, when compared to patients who underwent angioplasty, patients undergoing stent procedures showed better walking.	Nitinol stents are better options compared to bypass procedures for severe ischemic lower limbs. Furthermore, patients with cardiovascular comorbidities and with unfavorable anatomy are at an increased risk of surgical complications. The use of nitinol stents thus serve as a more practicable procedure compared to surgical revascularization.
10	Chiu et al. [62]	UK	(n=5,738)	29 studies (5738 patients) were reviewed for Aortofemoral bypass (AFB) and 11 studies (1490 patients) for aorto-iliac endarterectomy (AIE). Mortality rates for AFB was 4.1% vs 2.7% for AIE (p<0.0001). Morbidity rates were 16% for AFB and 12.1% for AIE.	Lower morbidity and mortality rates were seen after performing endarterectomy compared to bypass procedures.
11	DeCarlo et al. [63]	USA	(n=4,478)	Overall complications were much higher in patients with vein or prosthetic graft compared to patients who had a hybrid revascularization (OR = 1.45; 95% CI: 1.04-2.02; P = 0.028).	Patients with ICD underwent more complications post open bypass as opposed to patients who sought a hybrid approach to their treatment.
12	Kuma et al. [64]	Japan	Retrospective study (n=111)	75 patients (n=111) with ICD were compared to 43 patients with CLI. All patients had pre existing CFA stenosis and were undergoing CFAE. The 1- and 5-year primary patency rates were 100% and 100% for claudication and 95% and 95% for CLI, respectively.	Overall, with successful patency rates CFAE serves as a practical treatment option for patients.

While preventative measures and routine care of the extremities serve as effective methods to limit disease progression, a holistic approach must be considered depending on the patient profile. We looked at over 12 primary research articles and recognized that endovascular interventions are necessary to provide comprehensive treatment for PAD [53-64]. The findings are summarized in Table 2. According to the American Heart Association/American College of Cardiology, endovascular interventions serve as the optimal treatment for patients with lifestyle-limiting claudication as well as aortoiliac occlusive disease [64-66]. A similar review conducted by Klein et al. concluded that underused comprehensive treatment methods have resulted in subpar outcomes in patients. Patients with lifestyle-limiting claudication who failed to respond to non-invasive methods of treatment should be advised to undergo endovascular treatment, as it is far less invasive compared to surgical revascularization [67,68]. Endovascular intervention is appropriate for patients with femoropopliteal disease [68]. Schulte et al. found that no significant improvement was noticed after performing endovascular treatments on patients with infrapopliteal disease. The study was conducted as a multicenter randomized trial [69]. This conclusion was also derived from other studies, which observed that endovascular interventions for infrapopliteal disease did not show symptom alleviation [70]. Lastly, endovascular treatments should not be performed solely to prevent the progression of the disease to critical limb ischemia [66]. Table 2 summarizes the specialized endovascular techniques.

**Table 2:** Specialized endovascular techniques. DCB: drug-coated balloons; PCB: paclitaxel-coated balloons; IVL: intravascular lithotripsy; ePTFE: expanded polytetrafluoroethylene.

Technique	Procedure
Balloon angioplasty	This method is typically used for stenotic arteries or occlusions to blood flow. The minimally invasive procedure involves separating the media from the intima. Care should be taken to dilate the balloon intermittently to avoid impediments. A stent may be applied after balloon insertion to avoid further complications [71].
DCB	Balloons coated with paclitaxel are used to avoid the chances of restenosis as well as prevention of hyperplasia of the intima [72]. Albashaireh et al. found that PCB were highly effective for femoropopliteal lesions. 1609 subjects were identified who were treated with PCB and it significantly reduced the need for revascularization (RR 0.33, 95% CI 0.222–0.49, p < 0.001) as well as decreased the risk for bilateral restenosis (RR 0.33, 95% CI 0.222–0.49, p < 0.001) [73].
IVL balloons	Lithotripsy coated balloons are effectively used for managing severely calcified plaques. The procedure involves inducing shockwaves in severely calcified plaques to create breakage, making it effective to manage severely calcified femoropopliteal plaques. [74] In their study, Tepe et al. found that 153 patients who underwent IVL when compared to patients who underwent PBA showed more successful rates for the procedure (65.8% vs. 50.4%; p = 0.01) [74].
Balloon expandable stents	Stents are used to prevent intimal flaps and circumvent collapsing of the artery. Bare metal stents usually comprise nitinol or stainless steel whereas covered stents are made up of expanded polyfluoroethylene (ePTFE). Balloon expandable stents, for example, can be used for aorto-iliac disease. Balloon expandable stents can be of covered or bare metal type and both can be used to prevent intimal hyperplasia [72].

Combined endovascular interventions such as angioplasty, DCBs, balloon expandable stents, and lithotripsy have shown minimal restenosis rates and are favored over single treatments [71-74]. Although minimally invasive, endovascular interventions can result in complications, either after device insertion or during the procedure. Embolization is another feared complication of endovascular interventions. Patients with renal failure or other high-risk patients should opt for surgical methods instead, as emboli formation results are associated with high morbidity and mortality rates [72]. Hematomas can occur after percutaneous interventions, with retroperitoneal hematomas being a feared complication if the puncture site is above the inferior epigastric artery. Retroperitoneal hematomas can be repaired using endovascular techniques such as stent graft placement [75]. Arterial dissections and perforations are also complications but can be managed conservatively [72]. Acute kidney injury (AKI) is a frequently encountered systemic complication when using endovascular techniques due to the significant use of dyes. The use of contrast dyes for infrapopliteal interventions should be limited to 20 to 30 cc to avoid nephrotoxicity. This can be achieved by diluting the dye and reducing repeat injections. Patients with chronic kidney disease (CKD) may benefit from using CO_2_ as an additive for arterial imaging of the lower extremities [76].

Aorto-Iliac Artery

Aortoiliac disease (AID), also known as Leriche syndrome, involves the formation of occlusive atherosclerotic plaques at the infrarenal aorta, bifurcation of the aorta, or at the iliac arteries bilaterally. This causes a triad of symptoms such as bilateral thigh or hip pain, erectile dysfunction, and decreased femoral pulses [77-79]. Endovascular interventions for aortoiliac disease have shown low morbidity and mortality rates, making them favorable as a first-line intervention [68]. Vallabhaneni et al. looked at 14 patients with concomitant iliac obstruction and abdominal aortic aneurysm (AAA) over a period of 28 months who underwent endovascular aneurysm repair procedures (EVAR). Results suggested a 100% patency rate with limited length of stay in the hospital as well as subjective symptomatic improvement and no mortality rates [80]. With advancements in endovascular device profiles as well as enhanced experience in performing such procedures, this minimally invasive method is preferred over surgical procedures as it provides minimal complication rates as well as lower mortality rates [81]. However, a large-scale meta-analysis looked at eleven observational studies and used a sample of 4030 patients to identify the differences in success rates between direct surgical (DS) and endovascular hybrid (EH) revascularization. The study concluded that DS revascularization provided longer patency rates compared to EH interventions. Risk factors such as diabetes, hyperlipidemia, smoking, etc., were comparable in both groups; however, the age group for DS revascularization was significantly lower, which might have influenced the outcome [82]. A similar study conducted by Indes et al. observed the difference in patency rates between open-bypass and endovascular interventions in 5538 patients. The five-year patency rates between open-bypass versus endovascular revascularization were 82.7% vs. 71.4%, respectively [83].

According to the Trans-Atlantic Inter-Society Consensus II (TASC II), arterial lesions are classified from A to D depending on their anatomic distribution, nature of the lesion, and their response to endovascular or surgical methods [51]. TASC II A/B lesion types should be treated by endovascular methods. A plethora of studies show that endovascular interventions can also be used for type C/D lesions [68,84-86]. We looked at three meta-analyses that concluded that covered stents or kissing stents had a minimum of two-year patency rates in TASC II type C and D lesions and were proven to be safe, providing minimum complication rates as well as a shorter length of hospitalization [87-89]. Bekken et al. looked at 19 research articles and concluded that covered stents (CS) provided higher patency rates compared to bare metal stents (BMS) in TASC II C/D lesions [89]. Mwipatayi et al. also noted similar findings and came to the conclusion that CS demonstrated superior results in type C lesions, with lower rates of revascularization procedures required and higher patency rates when compared to BMS [90,91]. Additionally, findings reported by Klein et al. noted that the restenosis rate using CS was notably lower than that of BMS in type C and D lesions (HR, 0.136; 95% CI 0.042-0.442), while CS and BMS did not show significant differences in type A and B lesions (HR, 0.748; 95% CI 0.235-2.386) [68]. Embolization of the adjacent mesenteric and renal arteries can occur during aorto-iliac endovascular interventions. This is a feared complication and can be fatal. High-risk patients should opt for surgical methods, as thrombolysis or thrombectomy may not be successful in removing the embolus [72].

Common Femoral Artery

Surgery, particularly common femoral endarterectomy (CFAE), is the usual method of treating CFA stenosis. Due to its location, endovascular interventions for CFA stenosis are not a preferred option. In addition to the high restenosis rate [61], balloon angioplasty in such a location can be associated with complications such as dissection, which may require stenting [92]. Additionally, stents have an increased risk of breaking due to the anatomical site of placement. The possibility of restenosis and subpar outcomes may curtail future surgical interventions, which results in overall unfavorable outcomes [92]. Although surgical procedures provide long-term patency rates, endovascular interventions have been effective in reducing complications and providing better outcomes in the short term [68,92].

Femoropopliteal Artery

Endovascular interventions for the femoropopliteal segment have yielded suboptimal results in the past. Due to their location, restenosis occurrence, and low long-term patency rates, endovascular interventions have faced a distinctive problem [68]. Management of femoropopliteal artery occlusions can be classified according to the length of the lesion. Any lesion less than 5 cm that restricts blood flow can be treated using plain balloon angioplasty (PBA) [68,72].

Stent Grafts

Stent placement can be considered a feasible option for patients with lesions ranging from 5 to 15 cm. In their study, Schillingeret et al. found the restenosis rate of the superficial femoral artery to be much lower in patients with stents than in patients who have undergone angioplasty (24% and 43%, respectively) [61]. The incidence of in-stent restenosis (IRS) is highly common, and once it occurs, it is highly resistant to treatment [68,93]. That being said, there are more technical advancements in stent grafts that yield better results. For example, using nitinol stents instead of stainless steel stents yielded better results [61]. The use of self-expanding ePTFE grafts for femoropopliteal lesions has been indicated due to their resistance to growth [68]. Schillinger et al. assigned 104 patients with chronic limb ischemia and claudication to either stent therapy or PBA. Fifty-one patients who underwent stent procedures had a restenosis rate of 24% after six months versus patients who underwent PBA, who observed a restenosis rate of 42% (P=0.05) [61].

Re-Entry Catheters

With newer endovascular technologies at the forefront, the approach to treating femoropopliteal lesions is changing. A retrograde approach has yielded positive results [68]. Complex femoropopliteal lesions have shown successful results after being treated by a retrograde approach or re-entry catheters [94]. A retrospective single-center study conducted by Bausback et al. found that the outback catheter used in 113 patients showed successful recanalization in 90.7% (107/118) of the patients. Secondary patency rates in patients undergoing re-entry catheter methods were 89.1%. Overall, this approach exhibited successful results; however, the probability of restenosis was markedly high [95]. These findings are consistent with those found by Cannavale et al., where it was reported that the use of re-entry devices using the retrograde approach for patients with CTO in the femoropopliteal segment yielded >80% success rates [60]. While limitations such as high cost rates are identified with the use of catheters, more advancements can be expected in the endovascular armamentarium that would improve the management of patients with PAD.

Infra-Popliteal Arteries

Infrapopliteal interventions have been reserved for patients with CLI or rest pain. Often, patients with infrapopliteal lesions have to undergo multilevel revascularizations [96,97]. There is ongoing research but no conclusive findings about the end result of endovascular versus surgical treatment; however, a general consensus is to always begin with an endovascular intervention [96-98]. Balloon angioplasty is the most common method of approaching infrapopliteal lesions. The benefits of DCBs have been compared to balloon angioplasty; however, large-scale multicenter studies need to be conducted in order to understand the advantages of DCBs over balloon angioplasty [68,72]. Drug-eluting stents (DES) have shown positive results for focal infrapopliteal lesions compared to DCB or balloon angioplasty. In a database review conducted by Rastan et al., it was observed that using DES (mostly sirolimus-eluting) provided superior results compared to any other approach (the one-year primary patency rate was 86% ± 5%, while the mean target lesion revascularization rate and limb salvage rate were 9.9% ± 5% and 96.6% ± 4%, respectively [99]. Similarly, a meta-analysis conducted by Liu et al. found visible short-term results after using DES. However, more research needs to be performed to evaluate the long-term benefits of DES over DCB/balloon angioplasty [100].

Surgical techniques

*Aorto-Iliac Artery* A surgical approach for AI is only considered after endovascular methods have failed. Open bypass surgery is also recommended if there are diffuse lesions throughout the aortoiliac segment [51]. Open surgery is recommended for patients after assessing their risk factors and evaluating the presence of any comorbidities. A strong contraindication would be if a patient is unable to undergo anesthesia [101]. The use of PTFE or Dacron conduits does not seem to influence the outcome. Typically, a retroperitoneal or transperitoneal approach is considered for an aortofemoral bypass. Under circumstances such as the presence of unfavorable anatomy, extra-anatomic bypasses such as the axillofemoral or femoral-femoral bypass may be considered. However, extra-anatomic bypass is almost never recommended due to its subpar results [51]. The five-year patency rate of aortofemoral bypass surgery is between 64% and 95%, with an 80% success rate in the procedure and up to ten years of symptomatic relief [102,103].

Common Femoral Artery

Endovascular revascularization has become a popular option for the treatment of PAD due to advancements in the endovascular armamentarium with fewer complications and a minimally invasive nature. Due to the location of the CFA, stent placement has been a reluctant method of treatment [104]. However, in their study, Gouëffic et al. found that hospitalization rates among patients undergoing stent placement were notably lower than those among people undergoing surgery (3.2 ± 2.9 days vs. 6.3 ± 3 days; p < 0.0001, respectively). Perioperative morbidity and mortality rates were also significantly lower in the stenting group [105]. Similar results have also been reported by Kuma et al., where the one-year and five-year patency rates for patients with CFA with claudication undergoing endarterectomy were 100% and 100%, respectively, and patients with CFA and CLI undergoing endarterectomy showed 95% and 95% success rates, respectively [64]. These endovascular advancements, therefore, show more victorious results compared to surgical procedures, making surgical revascularization an unfavorable choice.

Femoro-Popliteal Artery 

A meta-analysis conducted by van de Weijer et al. found that postoperative complications of the femoropopliteal segment after bypass surgery were 37%, while endovascular treatment was 17% [106]. However, the reintervention rates were reported to be higher in patients who have undergone endovascular treatment when compared to those who have undergone surgical bypass (25.2% vs. 17.0%, P = 0.012) [107]. Nevertheless, the loss of patency in both groups was statistically insignificant [107]. In spite of that, femoropopliteal bypass is the gold standard procedure for patients with complete occlusion or lesions that are long [106].

## Conclusions

Atherosclerosis is the leading cause of PAD. Since a significant population with PAD remains asymptomatic, management is highly dependent on the severity of the patient profile. Patients older than 40 years of age should be monitored for any underlying risk factors, and any remarkable findings on physical examination involving pain or impairment while ambulating must be noted. DSA is the gold standard for visualizing vascular abnormalities but comes with significant limitations such as invasiveness, underestimation of arterial stenosis, and exposure to ionizing radiation, which may lead to renal failure. CTA provides a lower radiation dose but subsequently lacks the ability to provide a flow profile. MRA provides better grading of the arterial lumen than DSA but has the same contraindications in addition to being incompatible with implanted devices. DUS proves to be the best preliminary tool to catch the disease in its early stages, as it is comparable to DSA but also cheaper and less invasive. Smoking cessation, supervised exercise therapy, and lowering blood pressure are some of the first approaches to managing disease progression. Studies have shown that patients with atherosclerotic disease, irrespective of their baseline LDL levels, did not experience any major adverse limb effects when treated with statin therapy. Endovascular treatments were proven to be significantly better than surgical revascularization options. The implementation of 2D fusion imaging has significantly reduced radiation doses and contrast dye requirements, emphasizing the evolving nature of endovascular methods. Octogenarian patients who have multiple comorbidities prefer endovascular treatments because they have demonstrated better cardiovascular and limb outcomes when treated with endovascular revascularization compared to surgical treatments. Increasing technological advancements in endovascular procedures have made their overall outcomes comparable to surgical procedures. Nitinol stents are a preferable option compared to bypass procedures for extreme cases of lower limb ischemia. Patients with cardiovascular comorbidities and unfavorable anatomy face an increased risk of surgical complications. Therefore, the use of nitinol stents serves as a more practical and feasible procedure for revascularization in such cases. In conclusion, the evidence supports the overall benefits of endovascular interventions over surgical revascularization. Surgical bypass remains the gold standard for complete lesions of the femoropopliteal artery.

## Appendices

**Table 3:** The advancements of endovascular methods.

	Author	Country	Design and study population	Findings	Conclusion
1.	Eleissawy et al. [53]	Egypt and Belgium	Prospective cohort study (n=53)	14.84% (n=53) who underwent endovascular treatment showed 92% limb salvage compared to 88% in the surgical group (P = 0.564). The endovascular group showed a lower length of stay compared to the surgical group (6.24 ± 0.37 vs. 1.84 ± 0.19 days, respectively, P = 0.001).	The endovascular group showed lower length of hospital stay compared to the surgical group, thereby resulting in lesser complications. Limb salvage rates are higher in the endovascular group. Overall, endovascular interventions have comparable patency rates and lesser long term complications.
2.	Nordanstig et al. [54]	Sweden	Prospective randomized control study (n=158)	50% patients (n=158) were assigned to invasive treatment strategies (INV). There was a notable difference in the intermittent claudication distance (ICD) when INV was compared to non-INV management (INV = +124m vs non-INV= +50M p=0.003). Overall vascular quality of life (VascuQoL) also suggested results favoring INV methods (P≤0.001).	Overall improvements were observed in patients who underwent INV when compared to non-invasive methods. INV strategies provided better long-term outcomes and patients with ICD showed guaranteed improvements with revascularization.
3.	Caradu et al. [55]	UK	Retrospective non-randomized trial (n=255)	48% (n=255) patients underwent endovascular treatment using 2D fusion imaging. The injected contrast medium was significantly lower compared to the control group (34.7 ± 13.8 mL vs 51.3 ± 26.7 mL; P < 0.001) and dose-area product (8.9 ± 9.9 Gy/cm^2^ vs 13.5 ± 14.0 Gy/cm^2^; P = 0.003)	With advancements in endovascular interventions, an overall improvement in the quality of life can be expected in the patient. The use of 2D fusion imaging has caused >30% reduction in the radiation dose and contrast dye needed, which denotes the significance of overall evolution of endovascular methods.
4.	Fakhry et al. [56]	Netherlands	Randomized clinical trial (n=212)	106 patients (n=212) who underwent combination therapy (ER + SET) exhibited better outcomes than patients only on SET (from 117 m to 1237 m for an improvement of 1120 m vs from 135 m to 847 m for improvement of 712 m, respectively) (mean difference, 408 m; 99% CI, 195-622 m) as well as combination therapy showed better VascuQoL outcomes compared to SET (1.34 [99% CI, 1.04-1.64] in the combination therapy group vs 0.73 [99% CI, 0.43-1.03]).	Patients with ICD show comprehensive QoL improvements when SET is combined with ER. An integrated approach in the management of symptoms has proven to show better outcomes.
5	Totić et al. [57]	Bosnia and Herzegovina	Prospective study (n=80)	Patients with CLI and TASC II C and D lesions were followed for a period of 28 months. Overall survival rates in patients who sought surgical and endovascular treatment did not show significant differences (100% vs. 97.5%; p=0.317), respectively.	Endovascular treatments are preferred over surgical interventions as they are less invasive.
6	Pacha et al. [58]	USA	NIS DATABASE (n=394,504)	56.1% patients (n=394,504) patients underwent ER and showed lower in-hospital mortality rates compared to the surgical group (3.0% vs 5.8%, adjusted OR: 0.61 [95% CI: 0.58 to 0.63]) as well as lesser leg amputations compared to the surgical group (3.3% vs 4.1%, adjusted OR: 0.77 [95% CI: 0.74 to 0.79]).	Octogenarian patients showed better cardiovascular and limb outcomes when treated with ER compared to surgical treatments.
7	van den Hondel et al. [59]	Netherlands	Systematic review	A total of 13 articles were reviewed and it was deduced that endoprosthesis had similar outcomes compared to surgical bypass (31% vs. 55%, P=0.048, respectively) as well as fewer complications.	With new technological advancements in endovascular procedures, overall outcomes of patients are comparable with surgical procedures, with fewer obstacles.
8	Cannavale et al. [60]	UK and China	Retrospective study (n=32)	32 vessels were treated, of which 21 lesions 65.6% totally occluded. Recanalization with guidewire was successful in 71% (21 lesions) and usage of catheter alone yielded 29% (9 lesions) successful findings.	Demographic observations show that patients with complete total occlusions (CTO) of vessels usually are elderly and have comorbidities such as diabetes and hypertension, making surgical options unsafe. Endovascular procedures such as cross-catheters help in treating CTOs without significant problems.
9	Schillinger et al. [61]	Austria	Randomized controlled trial (n=104)	51 out of 104 patients underwent primary stent therapy and saw the rate of restenosis at 6 months was 24%. Additionally, when compared to patients who underwent angioplasty, patients undergoing stent procedures showed better walking.	Nitinol stents are better options compared to bypass procedures for severe ischemic lower limbs. Furthermore, patients with cardiovascular comorbidities and with unfavorable anatomy are at an increased risk of surgical complications. The use of nitinol stents thus serve as a more practicable procedure compared to surgical revascularization.
10	Chiu et al. [62]	UK	(n=5,738)	29 studies (5738 patients) were reviewed for aortofemoral bypass (AFB) and 11 studies (1490 patients) for aorto-iliac endarterectomy (AIE). Mortality rates for AFB was 4.1% vs 2.7% for AIE (p<0.0001). Morbidity rates were 16% for AFB and 12.1% for AIE.	Lower morbidity and mortality rates were seen after performing endarterectomy compared to bypass procedures.
11	DeCarlo et al. [63]	USA	(n=4,478)	Overall complications were much higher in patients with vein or prosthetic graft compared to patients who had a hybrid revascularization (OR = 1.45; 95% CI: 1.04-2.02; P = 0.028)	Patients with ICD underwent more complications post open bypass as opposed to patients who sought a hybrid approach to their treatment.
12	Kuma et al. [64]	Japan	Retrospective study (n=111)	75 patients (n=111) with ICD were compared to 43 patients with CLI. All patients had pre existing CFA stenosis and were undergoing CFAE.The 1- and 5-year primary patency rates were 100% and 100% for claudication and 95% and 95% for CLI, respectively.	Overall, with successful patency rates CFAE serves as a practical treatment option for patients.

**Table 4:** The specialized endovascular techniques. DCB: drug-coated balloons; PCB: paclitaxel-coated balloons; IVL: intravascular lithotripsy; ePTFE: expanded polytetrafluoroethylene.

Technique	Procedure
Balloon angioplasty	This method is typically used for stenotic arteries or occlusions to blood flow. The minimally invasive procedure involves separating the media from the intima. Care should be taken to dilate the balloon intermittently to avoid impediments. A stent may be applied after balloon insertion to avoid further complications [71].
DCB	Balloons coated with paclitaxel are used to avoid the chances of restenosis as well as prevention of hyperplasia of the intima [72]. Albashaireh et al. found that PCB were highly effective for femoropopliteal lesions. 1609 subjects were identified who were treated with PCB and it significantly reduced the need for revascularization (RR 0.33, 95% CI 0.222–0.49, p < 0.001) as well as decreased the risk for bilateral restenosis (RR 0.33, 95% CI 0.222–0.49, p < 0.001) [73].
IVL balloons	Lithotripsy-coated balloons are effectively used for managing severely calcified plaques. The procedure involves inducing shockwaves in severely calcified plaques to create breakage, making it effective to manage severely calcified femoropopliteal plaques [74]. In their study, Tepe et al. found that 153 patients who underwent IVL when compared to patients who underwent PBA showed more successful rates for the procedure (65.8% vs. 50.4%; p = 0.01) [74].
Balloon expandable stents	Stents are used to prevent intimal flaps and circumvent collapsing of the artery. Bare metal stents usually comprise nitinol or stainless steel whereas covered stents are made up of expanded polyfluoroethylene (ePTFE) Balloon expandable stents, for example, can be used for aorto-iliac disease. Balloon expandable stents can be of covered or bare metal type and both can be used to prevent intimal hyperplasia [72].
